# Neural integrators for decision making: a favorable tradeoff between robustness and sensitivity

**DOI:** 10.1152/jn.00976.2012

**Published:** 2013-02-27

**Authors:** Nicholas Cain, Andrea K. Barreiro, Michael Shadlen, Eric Shea-Brown

**Affiliations:** ^1^Department of Applied Mathematics, University of Washington, Seattle, Washington; and; ^2^Department of Physiology and Biophysics, University of Washington, Seattle, Washington

**Keywords:** decision making, neural integrator

## Abstract

A key step in many perceptual decision tasks is the integration of sensory inputs over time, but a fundamental questions remain about how this is accomplished in neural circuits. One possibility is to balance decay modes of membranes and synapses with recurrent excitation. To allow integration over long timescales, however, this balance must be exceedingly precise. The need for fine tuning can be overcome via a “robust integrator” mechanism in which momentary inputs must be above a preset limit to be registered by the circuit. The degree of this limiting embodies a tradeoff between sensitivity to the input stream and robustness against parameter mistuning. Here, we analyze the consequences of this tradeoff for decision-making performance. For concreteness, we focus on the well-studied random dot motion discrimination task and constrain stimulus parameters by experimental data. We show that mistuning feedback in an integrator circuit decreases decision performance but that the robust integrator mechanism can limit this loss. Intriguingly, even for perfectly tuned circuits with no immediate need for a robustness mechanism, including one often does not impose a substantial penalty for decision-making performance. The implication is that robust integrators may be well suited to subserve the basic function of evidence integration in many cognitive tasks. We develop these ideas using simulations of coupled neural units and the mathematics of sequential analysis.

many decisions are based on the balance of evidence that arrives at different points in time. This process is quantified via simple perceptual discrimination tasks in which the momentary value of a sensory signal carries negligible evidence, but correct responses arise from summation of this signal over the duration of a trial. At the core of such decision making must lie neural mechanisms that integrate signals over time (Gold and Shadlen 2007; Wang 2008; Bogacz et al. 2006). The function of these mechanisms is intriguing, because perceptual decisions develop over hundreds of milliseconds to seconds, while individual neuronal and synaptic activity often decays on timescales of several to tens of milliseconds, a difference of at least an order of magnitude. A mechanism that bridges this gap is feedback connectivity tuned to balance, and hence cancel, inherent voltage leak and synaptic decay (Cannon and Robinson 1983; Usher and McClelland 2001).

The tuning required for a circuit-based or cellular mechanism to achieve this balance presents a challenge (Seung 1996; Seung et al. 2000), illustrated in Fig. 1*A*, *top*, via motion of a ball on a smooth energy surface. Here, the ball position *E*(*t*) represents the total activity of a circuit (relative to a baseline marked 0); momentary sensory input perturbs *E*(*t*) to increase or decrease. If decay dominates (Fig. 1*A*, *top right*), then *E*(*t*) always has a tendency to “roll back” to baseline values, thus forgetting accumulated sensory input. Conversely, if feedback connections are in excess, then activity will grow away from the baseline value (Fig. 1*A*, *top center*). If balance is perfectly achieved via fine tuning (Fig. 1*A*, *top left*), then temporal integration can occur. That is, inputs can then smoothly perturb network activity back and forth, so that the network state at any given time represents the time integral of past inputs.

**Fig. 1.**
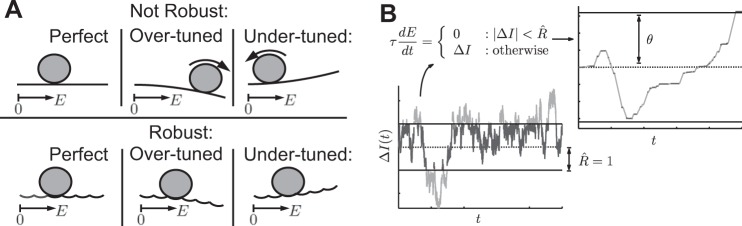
Schematic of neural integrator models. *A*: visualizing integration via an energy surface (Pouget and Latham 2002; Goldman et al. 2009). The robust integrator can “fixate” at a range of discrete values, indicated by a sequence of potential wells, despite mistuning of circuit feedback. These wells can be arbitrarily “close” in the energy landscape, providing a mechanism for graded persistent activity. Without these wells (the nonrobust case), activity in a mistuned integrator would either exponentially grow or decay, as at *top*. Perturbing the robust integrator from one well to the next, however, requires sufficiently strong momentary input. *B*: as a consequence, low-amplitude segments in the input signal Δ*I*(*t*), below a robustness limit *R*, are not accumulated by a robust integrator: only the high-amplitude segments are. The piecewise definition of *Eq. 5* captures this robustness behavior, resulting in the accumulated activity shown, and may be related to, e.g., a detailed bistable-subpopulation model. A decision is expressed when the accumulated value *E*(*t*) crosses the decision threshold θ.

Koulakov et al. (2002) proposed an alternative, circuit-based model, equivalent to movement along a scalloped energy surface made up of neighboring “energy wells” (Fig. 1*A*, *bottom*) (Pouget and Latham 2002; Goldman et al. 2009; Fransén et al. 2006). Importantly, even without finely tuned connectivity, network states can hold prior values without decay or growth, allowing integration of inputs over time. Thus this mechanism is called a robust integrator. However, the energy wells imply a minimum input strength to transition between adjacent states, with inputs below this limit effectively ignored. Intriguingly, this loss of sensitivity is shared by a mechanism of integration that was discovered at the level of single cells. Egorov et al. (2002) observed integration and graded steady states in layer V cells of entorhinal cortex, and movement among these states was only driven by stimuli stronger than a certain limit [as modeled in Fransén et al. (2006); Loewenstein and Sompolinsky (2003)].

These studies point to a general issue affecting integrator mechanisms: a loss of sensitivity to weak inputs. As made explicit in Koulakov et al. (2002) and Goldman et al. (2003), this may arise in a tradeoff in which robustness to parameter mistuning is gained. Here, we abstract the underlying mechanisms and present a general theoretical analysis of the tradeoff between sensitivity and robustness for decisions based on integrated evidence. We find that the tradeoff is favorable: decision speed and accuracy are lost when the integrator circuit is mistuned, but this loss is partially recovered by making the network dynamics robust. Thus, although the robust integrator discards the weakest portions of the evidence stream, enough evidence is retained to produce decisions that are faster and more accurate than would occur with unchecked over- or under-tuning of feedback (Fig. 1*B*). The implication is that cellular or circuit-based robust integrators may be remarkably well suited to subserve a variety of decision-making computations.

## MATERIALS AND METHODS

### Model and Task Overview

To explore the consequences of the robust integrator mechanism for decision performance, we begin by constructing a two-alternative decision-making model similar to that proposed by Mazurek et al. (2003). For concreteness, we concentrate on the forced choice motion discrimination task (Roitman and Shadlen 2002; Mazurek et al. 2003; Gold and Shadlen 2007; Churchland et al. 2008; Shadlen and Newsome 1996, 2001). Here, subjects are presented with a field of random dots, of which a subset move coherently in one direction; the remainder are relocated randomly in each frame. The task is to correctly choose the direction of coherent motion from two alternatives (i.e., left vs. right).

**Fig. 2.**
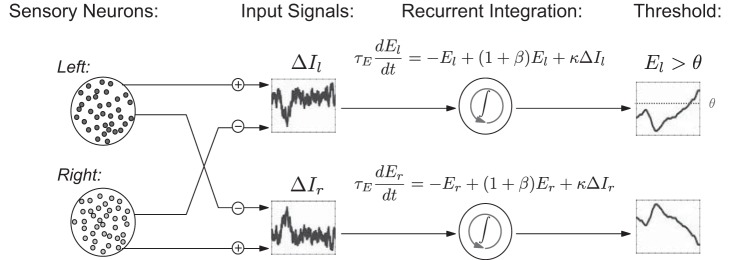
Overview of model. Simulations of sensory neurons and neural recordings are used to define the left and right inputs Δ*I*_l_(*t*) and Δ*I*_r_(*t*) to neural integrators. These inputs are modeled by Gaussian [Ornstein-Uhlenbeck (OU)] processes, which capture noise in the encoding of the motion strength by each pool of spiking neurons (see *Eqs. 1–3* for definition of input signals). Similar to Mazurek et al. (2003), the activity levels of the left and right integrators *E*_l_(*t*) and *E*_r_(*t*) encode accumulated evidence for each alternative. In the reaction time task, *E*_l_(*t*) and *E*_r_(*t*) race to thresholds to determine choice on each trial. In the controlled duration task, the choice is made in favor of the integrator with higher activity at the end of the stimulus presentation.

As in Mazurek et al. (2003) [see also Smith (2010)], we first simulate a population of neurons that represent the sensory input to be integrated over time. This population is a rough model of cells in extrastriate cortex (area MT) that encode momentary information about motion direction (Britten et al. 1992, 1993; Salzman et al. 1992). We pool spikes from model MT cells that are selective for each of the two possible directions into separate streams, labeled according to their preferred “left” and “right” motion selectivity (see Fig. 2). Two corresponding integrators then accumulate the difference between these streams, left-less-right or vice versa. Each integrator therefore accumulates the evidence for one alternative over the other. The dynamics of these integrators embody the tradeoff between robustness and sensitivity that is the focus of our study (see *Neural Integrator Model and the Robustness-Sensitivity Tradeoff*).

Depending on the task paradigm, different criteria may be used to terminate accumulation and give a decision. In the reaction time task, accumulation continues until activity crosses a decision threshold: if the leftward evidence integrator reaches threshold first, a decision that overall motion favored the leftward alternative is registered. In a second task paradigm, the controlled duration task, motion viewing duration is set in advance by the experimenter. A choice is made in favor of the integrator with greater activity at the end of the stimulus duration.

Accuracy is defined as the fraction of trials that reach a correct decision. Speed is measured by the time taken to cross threshold starting from stimulus onset. Reaction time (RT) is then defined as the time until threshold (decision time) plus 350 ms of nondecision time, accounting for other delays that add to the time taken to select an alternative [e.g., visual latencies, or motor preparation time, cf. Mazurek et al. (2003) and Luce (1986)]. The exact value of this parameter was not critical to our results. Task difficulty is determined by the fraction of coherently moving dots *C* (Britten et al. 1992; Mazurek et al. 2003; Roitman and Shadlen 2002). Accuracy and reaction time across multiple levels of task difficulty define the accuracy and chronometric functions in the reaction time task and together can be used to assess model performance. When necessary, these two numbers can be collapsed into a single metric, such as the reward per unit time or reward rate. In the controlled duration task, the only measure of task performance is the accuracy function.

### Sensory Input

**Fig. 3.**
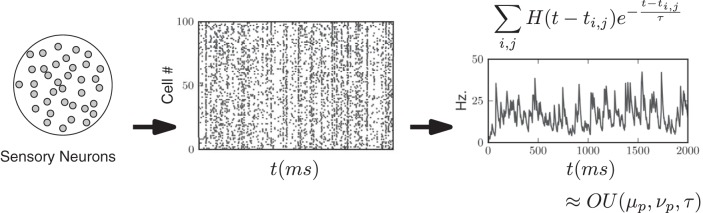
Construction of Gaussian (OU) processes to represent fluctuating, trial-by-trial firing rate of a pool of weakly correlated MT neurons (Bair et al. 2001; Zohary et al. 1994). As in Mazurek and Shadlen (2002), these motion sensitive neurons provide direct input to our model integrator circuits. Simulated spike trains from weakly correlated, direction selective pools of neurons are shown as a rastergram. All spikes before time *t*, a sum over the *j*th spike from the *i*th neuron, for all *i* and *j*, are convolved with an exponential filter, and then summed to create a continuous stochastic output (*right*); here, *H*(*t*) is the Heaviside function. We approximated this output by a simpler Gaussian (OU) process to simplify numerical and analytical computations that follow.

We now describe in detail the signals that are accumulated by the integrators corresponding to the left and right alternatives. First, we model the pools of leftward or rightward direction-selective sensory (MT) neurons as 100 weakly correlated [Pearson's correlation ρ = 0.11 (Zohary et al. 1994; Bair et al. 2001)] spiking cells (see Fig. 3). As in Mazurek and Shadlen (2002), neural spikes are modeled via unbiased random walks to a spiking threshold, which are correlated for neurons in the same pool. Increasing the variance of each step in the random walk increases the firing rate of each model neuron; it was therefore chosen at each coherence value to reproduce the linear relationship between coherence *C* and mean firing rate μ_l,r_ of the left and right selective neurons observed in MT recordings:

(1) $$\mu_{\text{l},\text{r}}\left(C\right)=r_{0}+b_{\text{l},\text{r}}C.$$

Here the parameters *r*_0_, *b*_l_, and *b*_r_ approximate recordings from MT (Britten et al. 1993); *r*_0_ = 20, and if evidence favors the left alternative, *b*_l_ = 0.4 and *b*_r_ = −0.2; if the right alternative is favored, these values are exchanged.

Next, the output of each spiking pool was aggregated. Each spike emitted from a neuron in the pool was convolved with an exponential filter with time constant 20 ms, an approximate model of the smoothing effect of synaptic transmission. These smoothed responses were then summed to form a single stochastic process for each pool [see Fig. 3, *right*, and Smith (2010)].

We then approximated the smoothed output of each spiking pool by a simpler stochastic process that captures the mean, variance, and temporal correlation of this output as a function of dot coherence. We used Gaussian processes *I*_l_(*t*) and *I*_r_(*t*) for the rightward- and leftward-selective pools (see Fig. 3). Specifically, we chose Ornstein-Uhlenbeck (OU) processes, which are continuous Gaussian processes generated by the stochastic differential equations

(2) $$dI_{\text{l},\text{r}}=\frac{\mu_{\text{l},\text{r}}\left(C\right)-I_{\text{l},\text{r}}}{\tau }dt+\sqrt{\frac{2v_{\text{l},\text{r}}\left(C\right)}{\tau }dW_{t}}$$

with mean μ_l,r_(*C*) as dictated by *Eq. 1* and noise contributed by the Wiener process *W*_*t*_. The variance μ_l,r_(*C*) and timescale τ were chosen to match the steady-state variance and autocorrelation function of the smoothed spiking process. As shown in results, this timescale affects the speed and accuracy of decisions under robust integration.

Our construction so far accounts for variability in output from left vs. right direction selective neurons. We now incorporate an additional noise source into the output of each pool. These noise terms [η_l_(*t*) and η_r_(*t*), respectively] could represent, for example, neurons added to each pool that are nonselective to direction or intrinsic variability in the integrating circuit. Each noise source is modeled as an independent OU process with mean 0, timescale 20 ms as above, and a strength (variance) ν_γ_/2. This noise strength is a free parameter that we vary to match behavioral data (see *Parameterizing and Comparing the Robust Integrator Model with Behavioral Data* and Fig. 15). We note that previous studies (Shadlen et al. 1996; Mazurek et al. 2003; Cohen and Newsome 2009) also found that performance based on the direction-sensitive cells alone can be more accurate than behavior and, therefore, incorporated variability in addition to the output of left and right direction selective MT cells.

Finally, the signals that are accumulated by the left and right neural integrators are constructed by differencing the outputs of the two neural pools:

(3) $$\begin{array}{c} \Delta I_{\text{l}}\left(t\right)=\left[I_{\text{l}}\left(t\right)+\eta_{\text{l}}\left(t\right)\right]-\left[I_{\text{r}}\left(t\right)+\eta_{\text{r}}\left(t\right)\right]\\ \Delta I_{\text{r}}\left(t\right)=-\Delta I_{\text{l}}\left(t\right). \end{array}$$

### Neural Integrator Model and the Robustness-Sensitivity Tradeoff

A central issue is the impact of variability in the relative tuning of recurrent feedback vs. decay in a neural integrator circuit. Below, we will introduce the mistuning parameter β, which determines the extent to which feedback and decay fail to perfectly balance. We first define the dynamics of the general integrator model on which our studies are based. This is described by the firing rates *E*_l,r_(*t*) of integrators that receive outputs from left-selective or right-selective pools *I*_l,r_(*t*) respectively. The firing rates *E*_l,r_(*t*) increase as evidence for the corresponding task alternative is accumulated over time:

(4) $$\begin{array}{c} \tau_{E}\frac{dE_{\text{l},\text{r}}}{dt}=-E_{\text{l},\text{r}}+\left(1+\beta \right)E_{\text{l},\text{r}}+\kappa \Delta I_{\text{l},\text{r}}\left(t\right)\\ =\beta E_{\text{l},\text{r}}+\kappa \Delta I_{\text{l},\text{r}}\left(t\right). \end{array}$$

The three terms in this equation account for leak, feedback excitation, and the sensory input (scaled by a weight κ = 1/9), with time constant τ_*E*_ = 20 ms. When the mistuning parameter β = 0, leak and self-excitation exactly cancel, and hence the integrator is perfectly tuned. An integrator with β ≠ 0 is said to be mistuned, with either exponential growth or decay of activity (in the absence of input). Imprecise feedback tuning is modeled by randomly setting β to different values from trial to trial (but constant during a given trial), with a mean value β̄ and a precision given by a standard deviation σ_β_. We assume that β̄ = 0 for most of the study. Thus the spread of β, which we take to be Gaussian, represents the intrinsic variability in the balance between circuit-level feedback and decay. Perfect tuning corresponds to σ_β_ = β̄ = 0, while σ_β_ ≠ 0 or β̄ ≠ 0 corresponds to a mistuned integrator. Finally, we set initial activity in the integrators to zero [*E*_l,r_(0) = 0], and impose reflecting boundaries at *E*_r_ = 0, *E*_l_ = 0 [as in, e.g., Smith and Ratcliff (2004)] so that firing rates never become negative.

The tradeoff between robustness to mistuning and sensitivity to inputs is described by the extended model

$$\tau_{E}\frac{dE_{\text{l},\text{r}}}{dt}=\{\begin{array}{ll} 0 & \left|\beta E_{\text{l},\text{r}}+\kappa \Delta I_{\text{l},\text{r}}\right|\leq \kappa R\\ \beta E_{\text{l},\text{r}}+\kappa \Delta I_{\text{l},\text{r}} & \text{otherwise} \end{array}$$

All subsequent results are based on this simplified model, which captures the essence of the robust integration computation. The first line is analogous to the series of potential wells depicted in Fig. 1: if the sum of the mistuned integrator feedback and the input falls below the robustness limit *R*, the activity of the integrator remains fixed. If this summed input exceeds *R*, the activity evolves as for the nonrobust integrator in *Eq. 4*. To interpret the robustness limit *R*, it is convenient to normalize by the standard deviation of the input signal:

$$\hat{R}=\frac{R}{\sqrt{\text{Var}\left[\Delta I_{\text{l},\text{r}}\left(t\right)\right]}}\equiv \frac{R}{S}$$

where *S* = $\sqrt{\text{Var}\left[\Delta I_{\text{l},\text{r}}\left(t\right)\right]}$. In this way, *R̂* can be interpreted in units of standard deviations of input OU process that are “ignored” by the integrator. This yields the final expression, which we reproduce for convenience:

(5) $$\tau_{E}\frac{dE_{\text{l},\text{r}}}{dt}=\{\begin{array}{ll} 0 & \left|\beta E_{\text{l},\text{r}}+\kappa \Delta I_{\text{l},\text{r}}\right|\leq \hat{\kappa }S\hat{R}\\ \beta E_{\text{l},\text{r}}+\kappa \Delta I_{\text{l},\text{r}} & \text{otherwise} \end{array}$$

To summarize, *Eq. 5* defines a parameterized family of neural integrators, distinguished by the robustness limit *R̂* As *R̂* → 0, the model reduces to *Eq. 4*. When additionally β = 0, the (perfectly tuned) integrator computes an exact integral of its input: *Eq. 5* then yields *E*_l,r_(*t*) ∞ ∫_*t*_^0^Δ*I*_l,r_ (*t*′)*dt*′. We analyze this robust integrator model below.

### Computational Methods

Monte Carlo simulations of *Eq. 5* were performed using the Euler-Maruyama method (Higham 2001), with *dt* = 0.1 ms. For a fixed choice of input statistics and threshold θ, a minimum of 10,000 trials were simulated to estimate accuracy and reaction time values. In simulations where σ_β_ > 0, results were generated across a range of β-values and then weighted according to a normal distribution. The range of values was chosen with no less than 19 linearly spaced points, across a range of ±3 SD around the mean β̄. Simulations were performed on NSF Teragrid clusters and the UW Hyak cluster.

Reward rate values presented in *Reward Rate and the Robustness-Sensitivity Tradeoff* are presented as maximized by varying the free parameter θ; values were computed by simulating across a range of θ values. The range and spacing of these values were chosen dependent on the values of *R̂* and β for the simulation; the range was adjusted to capture the relative maximum of reward rate as a function of θ, while the spacing was adjusted to find the optimal θ value with a resolution of ±0.1: the values of θ and ν_γ_ (see table included in Fig. 15) were chosen to best match accuracy and chronometric functions to behavioral data reported in Roitman and Shadlen (2002). This was accomplished by minimizing the sum-squared error in data vs. model accuracy and chronometric curves across a discrete grid of θ and ν_γ_ values, with a resolution of 0.1.

Autocovariance functions of integrator input presented in *Analysis: Robust Integrators and Decision Performance* were computed by simulating an Ornstein-Uhlenbeck process using the exact numerical technique in Gillespie (1996) with *dt* = 0.1 ms to obtain a total of 227 sample values. Sample values of the process less than the specified robustness limit R̂ were set to 0, and the autocovariance function was computed using standard Fourier transform techniques.

## RESULTS

### How Do Robustness and Mistuning Affect Decision Speed and Accuracy?

A general issue affecting neural systems that integrate stimuli is the balance between mechanisms that lead their activity to decay vs. grow over time (Fig. 1*A*). If these are tuned to a perfect balance, the result is graded persistent activity that can accumulate and store inputs. Robust integrators provide an alternative to such fine tuning but at the cost of lost sensitivity to input signals, represented by the energy wells in Fig. 1*A*.

**Fig. 4.**
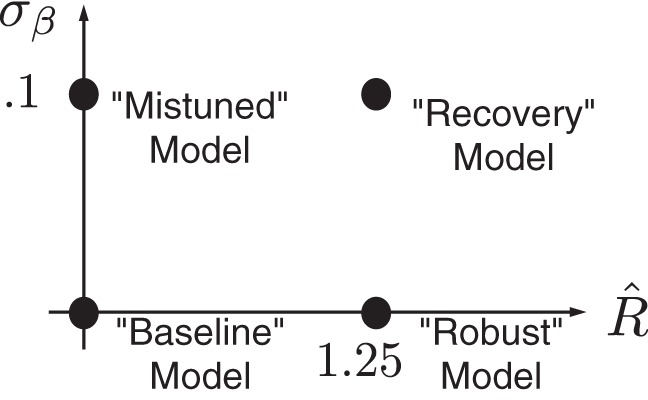
Parameter space view of 4 integrator models, with different values of the robustness limit *R̂* and feedback mistuning variability σ_β_. The impact of transitioning from one model to another by changing parameters is either to enhance or diminish performance or to have a neutral effect (see text).

Below we explore the costs and benefits of robust integration in terms of the general neural integrator model of *Eq. 5*. This model summarizes the underlying issues via two key parameters. The first, β, describes mistuning of the integrator away from “perfect” dynamics, so that its activity decays or grows autonomously (Fig. 1*A*). We describe the extent of mistuning by σ_β_, which represents the standard deviation of β from the ideal value β̄ = 0. The second key parameter is the robustness limit *R̂*. We emphasize dual effects of *R̂*: as *R̂* increases, the integrator becomes able to produce a range of graded persistent activity for ever-increasing levels of mistuning (see Fig. 1*A*, where *R̂* corresponds to the depth of energy wells). This prevents runaway increase or decay of activity when integrators are mistuned; intuitively, this might lead to better performance on sensory accumulation tasks. At the same time, as *R̂* increases, a larger proportion of the evidence fails to affect the integrator (see Fig. 1*B*, where *R̂* specifies a limit within which inputs are ignored). Such sensitivity loss should lead to worse performance. This implies a fundamental tradeoff between competing desiderata: *1*) one would prefer to integrate all relevant input, favoring small *R̂*, and *2*) one would prefer an integrator robust to mistuning (e.g., σ_β_ > 0), favoring large R̂. Thus it makes sense to assess the effect of robustness under different degrees of mistuning, as represented schematically in Fig. 4.

**Fig. A4.**
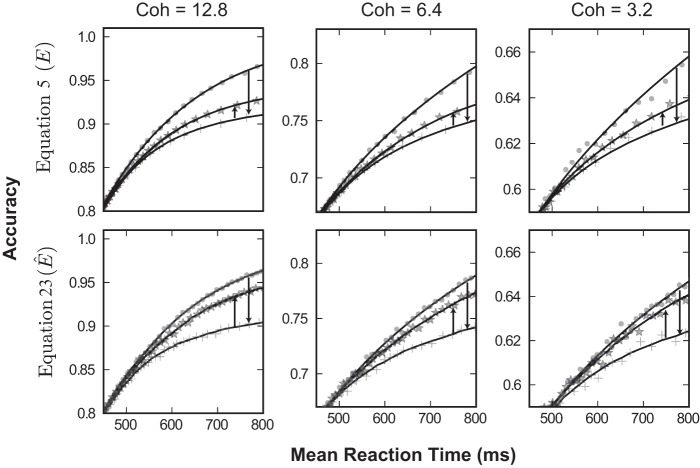
Performance comparison of 2 models of robust integration, reaction time task. In each panel, the lines are the same as in Fig. 6 legend. Downward arrows indicates the impact of mistuning: increasing σ_γ_ from 0 to 0.1. Upward arrows show the effect of robustness: increasing *R̂* from 0 to 1.25. Overall, we see that robustness has an even greater positive impact on the performance of the circuit-based model (here *q* = 1, *r*^−^ = 0, *r*^+^ = 50, *N* = 250, κ = 1/9; *p* is then adjusted to give the required robustness level). Data were generated from 50,000 numerical trials per value.

To assess this performance, we consider relationships between decision speed and accuracy in both controlled duration and reaction time tasks. In the controlled duration task, we simply vary the stimulus presentation duration and plot accuracy vs. experimenter-controlled stimulus duration. In the reaction time task, we vary the decision threshold θ, treated as a free parameter, over a range of values, thus tracing out the parametric curve for all possible pairs of speed and accuracy values. Here, speed is measured by reaction time (see materials and methods). For both cases, we use a single representative dot coherence (*C* = 12.8 in *Eq. 1*); similar results were obtained using other values for motion strength (see Fig. A4).

**Fig. 5.**
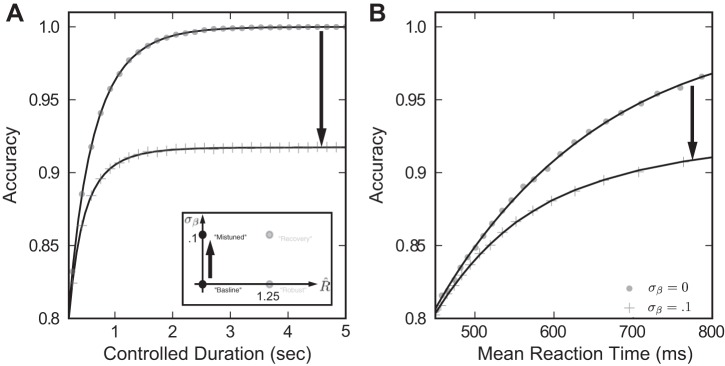
Mistuned feedback diminishes decision performance. *Inset*: plots depict a move in parameter space from the baseline model to the mistuned model by changing σ_β_ = 0 → 0:1. In this and subsequent plots, simulation results are given with markers; lines are rational polynomial fits. *A*: in the controlled duration task, accuracy is lower for the mistuned model than for the baseline model at every trial duration, indicating a loss of performance when σ_β_ increases. *B*: in the reaction time task, we parametrically plot all [reaction time (RT), accuracy] pairs attained by varying the decision threshold θ. Once again, accuracy is diminished by mistuning for a fixed mean reaction time.

We first establish the performance impact of mistuning in the absence of robustness. We begin with a case we call the “baseline” model (Fig. 4), for which there is no mistuning or robustness: σ_β_ = *R̂* = 0: Speed accuracy plots for this model are shown as filled dots in Fig. 5, *A* and *B*, for the controlled duration and reaction time tasks, respectively. We compare the baseline model with the “mistuned” model, indicated by crosses, for which the feedback parameter has a standard deviation of σ_β_ = 0.1 (i.e., 10% of the mean feedback) and robustness *R̂* = 0 remains unchanged. In the controlled duration task (Fig. 5*A*), we observe that mistuning diminishes accuracy by as much as 10%, and this effect is sustained even for arbitrarily long viewing windows (Usher and McClelland 2001; Bogacz et al. 2006). The same effect is apparent in the reaction time task (Fig. 5*B*): for a fixed reaction time, the corresponding accuracy is decreased.

**Fig. 6.**
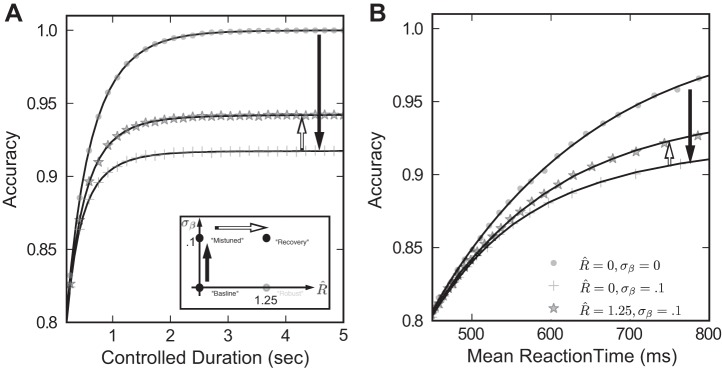
Increasing the robustness limit *R̂* helps recover performance lost due to feedback mistuning. Results of simulation are plotted with fitting lines. *Inset*: we illustrate this by moving in parameter space from the mistuned model to the recovery model, by changing *R̂* = 0 → 1.25. The impact on decision performance is shown for both the controlled duration (*A*) and reaction time (*B*) tasks. We find that *R̂* > 0 yields a performance gain for the recovery model compared with the mistuned model (i.e., for a fixed accuracy, mean reaction time is increased).

While maintaining feedback mistuning, we next increase the robustness limit to *R̂* = 1.25. We call this case the “recovery” model because robustness compensates in part for the performance loss due to feedback mistuning: the speed accuracy plots in Fig. 6 for the recovery case, indicated by stars, lie above those for the mistuned model. For example, at the longer controlled task durations (Fig. 6*A*) and reaction times (Fig. 6*B*) plotted, 30% of the accuracy lost due to integrator mistuning is recovered via the robustness limit *R̂* = 1.25. This degree of improvement underestimates the recovery attainable in some more realistic models. Indeed, a less simplified model of the integrator achieves a larger performance recovery (approaching ≈75%; see *Eq. 22* and Fig. A4).

**Fig. 7.**
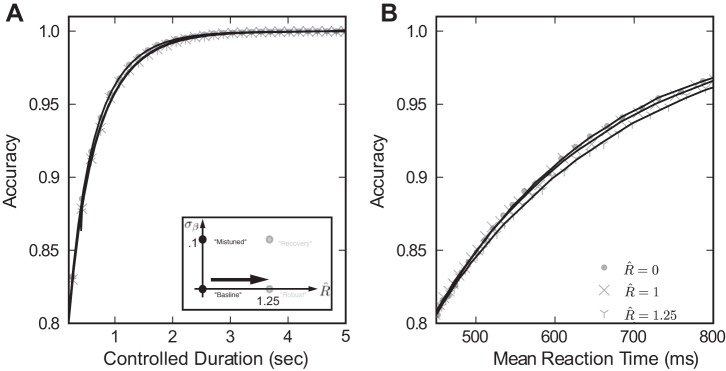
Increasing *R̂* alone does not compromise performance. Only simulation results, without fitting lines, are plotted for clarity. *Inset*: we illustrate this by moving in parameter space directly from the baseline to the “robust” model. For both controlled duration (*A*) and reaction time tasks (*B*), we plot the relationship between mean reaction time and accuracy. Circles give results for the baseline model, and “*x*” and “*y*” markers for the robust model at *R̂* = 1 and 1.25, respectively. These curves are very similar in the baseline case, indicating little change in decision performance due to the robustness limit *R̂* = 1.25.

One possible reason for the modest recovery of accuracy in Fig. 6 is that robustness itself reduces decision speed and accuracy. However, this does not appear to be a viable explanation. When the same degree of robustness accompanies a perfectly tuned integrator, the “robust” case in Fig. 4, there is negligible loss of performance. In particular, Fig. 7 demonstrates that when σ_β_ = 0, speed accuracy curves for *R̂* = 1.25 almost coincide with those for the baseline case of *R̂* = 0. We note that since *R̂* measures ignored input in units of the standard deviation, the integrator circuit disregards as much as 75% of the input stimulus at low coherence values. Given this large amount of ignored stimulus, the fact that the robust integrator produces nearly the same accuracy and speed as the baseline case is surprising, as one might expect ignoring stimulus to be detrimental to performance. This implies that the robust model can protect against feedback mistuning, without substantially sacrificing performance when feedback is perfectly tuned.

To summarize, the robust integrator appears well suited to the decision tasks at hand, countering some of the performance lost when feedback is mistuned. Moreover, even without mistuning, a robust integrator still performs as well as the baseline case that integrates all information in the input signal. In the next section, we begin to explain this observation by constructing several simplified models and employing results from statistical decision-making theory.

### Analysis: Robust Integrators and Decision Performance

#### Controlled duration task: temporally independent signals.

We can begin to understand the effect of the robustness limit on decision performance by formulating a simplified version of the evidence accumulation process. We focus first on the controlled duration task, where the analysis is somewhat simpler. Readers preferring a brief statement of the underlying mechanisms may skip to the *Summary of analysis* and proceed to the rest of the study from there.

Our first simplification is to consider a single accumulator *E*, which receives evidence for or against a task alternative. Moreover, we consider increments of evidence that arrive both independently and discretely in time. The value of *E* on the *i*th time step, *E*_*i*_, is allowed to be either positive or negative, corresponding to accumulated evidence favoring the leftward or rightward alternatives, respectively. On each time step, *E*_*i*_ increments by an independent, random value *Z*_*i*_ with a probability density function (PDF) *f*_*Z*_(*Z*). We first describe an analog of the baseline model above (i.e., in the absence of robustness, *R̂* = 0). Here, we take the increments *Z*_*i*_ to be independent, identical, and Gaussian distributed, with a mean μ > 0 (establishing the preferred alternative) and standard deviation σ: that is, *Z*_*i*_ ∼ *N* (μ, σ^2^). After the *n*th step, we have

$$E_{n}=\sum_{i=1}^{n}Z_{i}.$$

In the controlled duration task, a decision is rendered after a fixed number of time steps *N*, (i.e., *n* = *N*), and a correct decision occurs when *E*_*N*_ > 0. By construction, *E*_*n*_ ∼ *N* (*n*μ, *n*σ^2^), which implies that accuracy can be computed as a function of the signal-to-noise ratio (SNR) s=μ/σ of a sample:

(6) $$\text{Accuracy}=\int_{0}^{\infty }\frac{1}{\sqrt{2\pi N\sigma^{2}}}e^{-\frac{{\left(x-N\mu \right)}^{2}}{2N\sigma^{2}}}dx=\frac{1+\text{Erf}\left(\sqrt{\frac{N}{2}s}\right)}{2}.$$

**Fig. 8.**
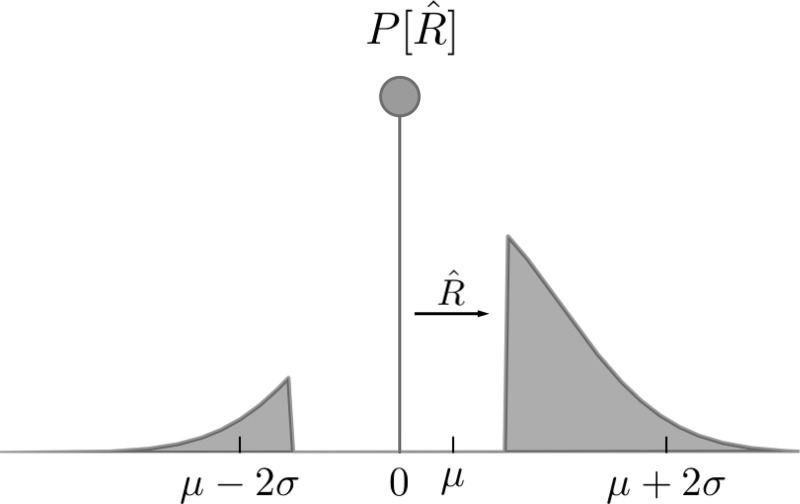
*R̂* affects the discrete time increment distribution. The probability density function of the random variable *Z_R̂_*, with probability mass for values between the robustness limit *R̂* reallocated as a delta function centered at zero (in this figure, *R̂* = 1).

Next, we change the distribution of the accumulated increments *Z*_*i*_ to construct a discrete time analog of the robust integrator. Specifically, increasing the robustness parameter to *R* > 0 affects increments *Z*_*i*_ by redefining the PDF *f*_*Z*_(*Z*) so that weak samples do not add to the total accumulated “evidence,” precisely as in *Eq. 5*. [Models where such a central “region of uncertainty” of the sampling distribution is ignored were previously studied in a race-to-bound model (Smith and Vickers 1989); see discussion]. This requires reallocating probability mass below the robustness limit to a weighted delta function at zero (Fig. 8*A*). Specifically:

(7) $$fz_{R}\left(Z\right)=\delta \left(Z\right)\int_{-R}^{R}fz\left(Z\prime \right)dZ\prime +\{\begin{array}{cc} 0 & \left|Z\right|<R.\\ fz\left(Z\right) & \text{otherwise} \end{array}$$

**Fig. 9.**
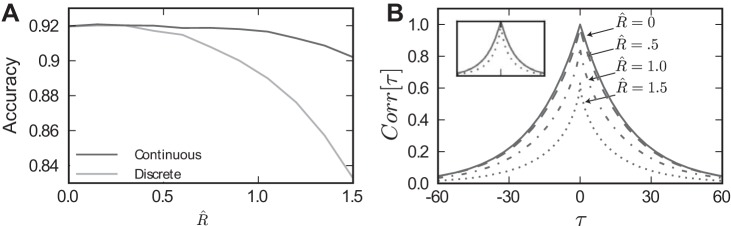
Accuracy of the discrete/independent and continuous/correlated models for the controlled duration task, *T* = 500 ms. *A*: approximation of the performance of the continuous/correlated model via *Eq. 15* is plotted as a black curve, and that predicted by the discrete/independent model with identical signal increments is plotted as a gray curve. *B*: disparity in performance of these 2 models can be partially understood by observing the decorrelating effect of *R̂* on the autocorrelation function for the evidence stream in the continuous/correlated model. *Inset*: 2 of these same functions (for *R̂* = 0 and *R̂* = 1.5) are plotted normalized to their peak value.

To estimate decision accuracy with robustness *R̂* > 0, we sum *N* random increments from this distribution forming the cumulative sum *E_N_R̂__*. As above, a correct decision occurs on trials where *E_N_R̂__* > 0. As shown in Fig. 9, the replacement of increments with zeros has negligible effect on accuracy when *R̂* is less than ∼0.5. For larger values of *R̂*, accuracy diminishes faster for the discrete/independent model (light curve) compared with the continuous/correlated model (dark curve). Loss of accuracy is expected for both models as robustness effectively prevents stimulus information from affecting the decision. However, the continuous model suffers less than the discrete approximation, owing to the one important difference: the presence of temporal correlations in the evidence stream. We will return to this matter below. First, we give an explanation for the negligible effect of *R̂* on decision accuracy for either model.

The central limit theorem allows us to approximate the new cumulative sum *E_N_R̂__* as a normal distribution (for sufficiently large *N*), with μ and σ in *Eq. 6* replaced by the mean and standard deviation of the PDF defined by *Eq. 7*. As before, we normalize *R* by the standard deviation of the increment, *R̂* = *R*/σ, and then express the fraction correct Accuracy*_R̂_* as a function of *R̂* and *s*. One can think of *R̂* as perturbing the original accuracy function given in *Eq. 6*. Although this perturbation has a complicated form, we can understand its behavior by observing that its Taylor expansion (see *Derivation of Eqs. 8 and 20* for more details) does not have first or second-order contributions in *R̂* :

(8) $${\text{Accuracy}}_{\hat{R}}\left(N\right)\approx \text{Accuracy}\left(N\right)-\frac{\sqrt{N}s\left(1+2s^{2}\right)e^{-\frac{{\left(1+N\right)}_{s}^{2}}{2}}}{6\pi }{\hat{R}}^{3}+O\left({\hat{R}}^{5}\right).$$

Thus, for small values of *R̂* (giving very small *R̂*^3^), there will be little impact on accuracy. *Equation 8* can therefore partially explain the key observation in Fig. 7*A* that *R̂* can be substantially increased while incurring very little performance loss.

Now we return to the comparison between the discrete/independent and continuous/correlated models, by setting the SNR of the sampling distribution identical to the steady-state distribution of the input signal to the neural integrator model (see *Sensory Input*). The interval between samples is set to match accuracy performance of the continuous time model at *R̂* = 0. The gray line in Fig. 9*A* shows the accuracy for the discrete time model, as the robustness limit *R̂* is increased. The discrete time model predicts a decrease in accuracy at *R̂* = 0.5; intriguingly, this is not seen in an analogous continuous time model. In the next sections, we first describe this continuous time model and explain how the discrepancy in the impact of robustness can be resolved by accounting for the temporally correlated structure of the continuous time signal.

#### Controlled duration task: temporally correlated signals.

We next extend the analysis of the controlled duration task in the previous section to treat integration of temporally correlated signals. In particular, we consider the continuous time signals described in *Sensory Input* above (although our conclusions would apply to temporally correlated processes in discrete time as well). We follow the method developed in Gillespie (1996) to describe the mean and variance of the integral of a continuous input signal. The challenge here lies in the temporal correlations in the Gaussian OU input signal (see *Sensory Input*). As in the previous section, we describe the distribution of the integrated signal at the final time *T*.

We first replace the discrete input samples *Z*_*i*_ from the previous section with a continuous signal *Z*(*t*), which we take to be a Gaussian process with a correlation timescale derived from our model sensory neurons (see materials and methods). We define the integrated process

(9) $$\frac{dE}{dt}=Z\left(t\right)\to E\left(t\right)=\int_{0}^{t}Z\left(t\prime \right)dt\prime$$

with initial condition *E*(0) = 0.

Assuming that *Z*(*t*) satisfies certain technical conditions that are easily verified for the OU process [wide-sense stationarity, α-stability, and continuity of sample paths (Gardiner 2002; Billingsley 1986; Gillespie 1996)], we can construct differential equations for the first and second moments 〈 *E*(*t*)〉 and 〈*E*^2^(*t*)〉 evolving in time. We start by taking averages on both sides of our definition of *E*(*t*) and, noting that *E*(0) = 0, compute the time-varying mean:

(10) $$\frac{d\langle E\left(t\right)\rangle }{dt}=\langle Z\left(t\right)\rangle \Rightarrow \langle E\left(t\right)\rangle =t\langle Z\left(t\right)\rangle .$$

Similarly, we can derive a differential equation for the second moment of *E*(*t*):

(11) $$\frac{d\langle E^{2}\left(t\right)\rangle }{dt}=2\langle Z\left(t\right)E\left(t\right)\rangle .$$

The right-hand side of this equation can be related to the area under the autocovariance function *A*(τ) ≡ 〈*Z*(*t*)*Z*(*t* + τ)〉 − 〈*Z*(*t*)〉^2^ of the process *Z*(*t*):

(12) $$\begin{array}{c} \langle Z\left(t\right)E\left(t\right)\rangle =\langle Z\left(t\right)\int_{0}^{t}Z\left(s\right)ds\rangle =\int_{0}^{t}\langle Z\left(t\right)Z\left(s\right)\rangle ds\\ =\int_{0}^{t}\langle Z\left(t\right)Z\left(t-\tau \right)\rangle d\tau \\ =\int_{0}^{t}A\left(\tau \right)+{\langle Z\left(t\right)\rangle }^{2}d\tau \end{array}$$

We now have an expression for how the second moment evolves in time. We can simplify the result via integration by parts and the fact that 〈*Z*(*t*)〉 is constant in time:

(13) $$\langle E^{2}\left(t\right)\rangle =2\int_{0}^{t}\int_{0}^{s}A\left(\tau \right)+{\langle Z\left(t\right)\rangle }^{2}d\tau ds=2\int_{0}^{t}\left(t-\tau \right)A\left(\tau \right)d\tau +t^{2}{\langle Z\left(t\right)\rangle }^{2}\Rightarrow \text{Var}\left[E\left(t\right)\right]=2\int_{0}^{t}\left(t-\tau \right)A\left(\tau \right)d\tau .$$

Because *E*(*t*) is an accumulation of Gaussian random samples *Z*(*t*), it will also be normally distributed and hence fully described by the mean (*Eq. 10*) and variance (*Eq. 13*) (Billingsley 1986).

To model a nonrobust integrator, we take *Z*(*t*) to be a OU process with steady-state mean and variance μ and σ^2^, and time constant τ. For the robust case, we can follow *Eq. 5* and parameterize a family of processes *Z*_*R̂*_(*t*) with momentary values below the robustness limit *R̂* set to zero. (Here, we again normalize the robustness limit by the standard deviation of the OU process.) We numerically compute the autocovariance functions *A*_*R̂*_(τ) of these processes and use the result to compute the required mean and variance, and hence time-dependent signal-to-noise ratio SNR(*t*), for the integrated process *E*(*t*). This yields

(14) $${\text{SNR}}_{\hat{R}}\left(t\right)=\frac{\langle E\left(t\right)\rangle }{\sqrt{Var\left[E\left(t\right)\right]}}=\frac{tE\left[Z_{\hat{R}}\left(t\right)\right]}{\sqrt{2\int_{0}^{t}\left(t-\tau \right)A_{\hat{R}}\left(\tau \right)d\tau }}.$$

Under the assumption that *E*(*T*) is approximately Gaussian for sufficiently long *T* (which can be verified numerically), we use this SNR to compute decision accuracy at *T*:

(15) $${\text{Accuracy}}_{\hat{R}}\left(T\right)\approx \frac{1+\text{Erf}\left(\frac{1}{\sqrt{2}}{\text{SNR}}_{\hat{R}}\left(T\right)\right)}{\text{2}}.$$

This function is plotted for *T* = 500 ms as the black line in Fig. 9*A*. The plot shows that accuracy remains relatively constant until the robustness limit *R̂* exceeds ≈1.25, a longer range of *R̂* values than for the discrete time case (compare gray curve vs. black curve in Fig. 9*A*).

Why does the robustness limit appear to have a milder effect on degrading decision accuracy for our temporally correlated vs. independent input signals? We can get some insight into the answer by examining the autocovariance functions *AR̂*(τ), which we present in Fig. 9*B*. When normalized by their peak value, the autocovariance for *R̂* > 0.5 falls off more quickly with respect to the time lag τ (see Fig. 9*B*, *inset*), indicating that subsequent samples become less correlated in time. Thus there are effectively more “independent” samples that are drawn over a given time range *T*, improving the fidelity of the signal and hence decision accuracy. Such an improvement clearly has no room to occur when samples are already independent, as in the preceding section. Interestingly, this argument, that, all else being equal, the sum of samples with lower correlation will yield better decision accuracy, is the same as that applied to samples pooled across neural populations by (Zohary et al. 1994; Averbeck et al. 2006).

**Fig. 10.**
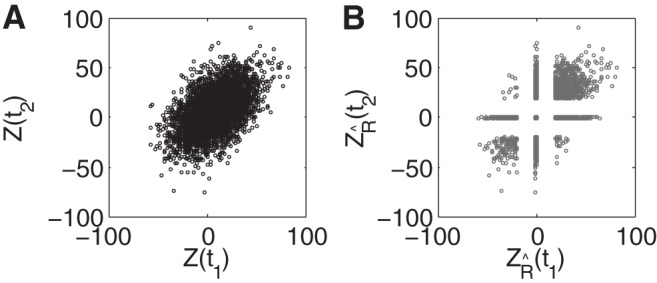
Distribution of 2 neighboring (in time) samples of the incoming signal. *A*: before application of the robustness operation, the samples *Z*(*t*_1_) and *Z*(*t*_2_) covary with correlation coefficient Corr = 0:5. *B*: after applying robustness (*R̂* = 1), samples with *Z*(*t*_1_) < *R̂* are mapped to *Z*_*R̂*_ (*t*_1_) = 0, and likewise for samples with *Z*(*t*_2_) < *R̂*. As a consequence, samples covary less in the region where either sample has been thresholded to zero, yielding Corr = 0:42.

The impact of the robustness limit on temporally correlated signals can be visualized by considering how robustness transforms a set of correlated (Gaussian) random variables, each representing the value of the signal at a nearby point in time (Fig. 10). As proven by (Lancaster 1957) and applied in a different context by (de la Rocha et al. 2007; Dorn and Ringach 2003), the nonlinear, thresholding action of this function must reduce the correlation of these variables; Fig. 10 demonstrates this explicitly. This is the mechanism behind the decrease in autocorrelation above.

As pointed out to us by a reviewer, the same setup leads to a complementary view of why the robustness operation can have minimal impact on decision accuracy. The more correlated a set of random samples is, the greater the chance that they will have the same deviation from the mean, i.e., that nearby samples in time will all provide evidence in favor of the same task alternative. The limited effect of robustness on decision accuracy suggests that, even if some of these samples are rectified to zero, other (correlated) samples remain that lead to the same final decision.

In summary, our analysis of decision performance for the controlled duration task shows that two factors contribute to the preservation of decision performance for robust integrators. The first is that the momentary SNR of the inputs is barely changed for robustness limits up to *R̂* ≈ 0.5. The second is that, as *R̂* increases, the signal *Z_R̂_*(*t*) being integrated becomes less correlated in time. This means that (roughly) more independent samples will arrive over a given time period.

#### Reaction time task.

In the context of threshold crossing in the reaction time task, the accumulation of increments toward decision thresholds can be understood as the sequential probability ratio test, where the log odds for each alternative are summed until a predefined threshold is reached (Wald 1945; Gold and Shadlen 2002; Luce 1963; Laming 1968). Wald (1944) provides an elegant method of computing decision accuracy and speed (reaction time). The key quantity is given by the moment generating function [MGF, denoted *M*_*Z*_(ω) below] for the samples *Z* [see Luce (1986), Link and Heath (1975), and Doya (2007)]. Under the assumption that thresholds are crossed with minimal overshoot of the accumulator on the final step, we have the following expressions:

(16) $$\text{Accuracy}=\frac{1}{1+e^{\theta h_{0}}}$$

(17) $$\text{RT}=\frac{\theta }{E\left[Z\right]}\text{tanh}\left[-\frac{\theta }{2}h_{0}\right]$$

where *h*_0_ is the nontrivial real root of the equation *M*_*Z*_(ω) = 1 and θ is the decision threshold.

We first consider the case of a nonrobust integrator, for which the samples *Z* are again normally distributed. In this case, we must solve the following equation to ω = *h*_0_:

(18) $$M_{Z}\left(\omega \right)=E_{z}\left[e^{\omega z}\right]=\int_{-\infty }^{\infty }f_{Z}\left(z\right)e^{\omega *z}dz=e^{\frac{\omega^{2}\sigma^{2}}{2}+\omega \mu }=1.$$

It follows that ω = 0 and ω = *h*_0_ = − 2 μ / σ^2^ provide the two real solutions of this equation. (Wald's Lemma ensures that there are exactly two such real roots, for any sampling distribution meeting easily satisfied technical criteria.)

**Fig. 11.**
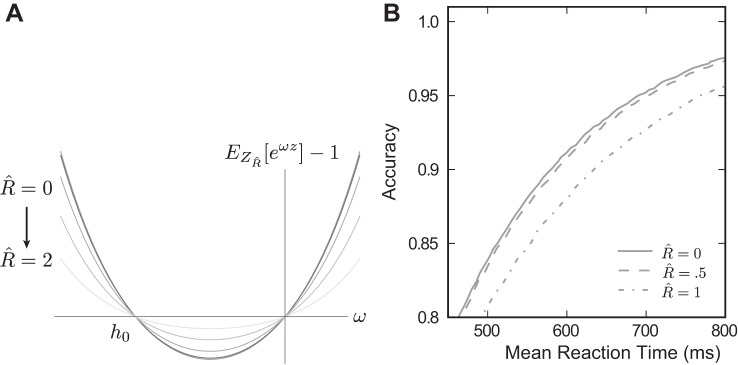
In the discrete model *R̂* increases RT but not accuracy. *A*: the second real root *h*_0_ of *M_Z_R̂__*(*s*) remains unchanged as *R̂* increases from 0 → 2 (lines are uniformly distributed in this range). This implies that in the reaction time task, no changes in the accuracy will be observed (see *Eq. 16*). *B*: however, the speed accuracy tradeoff will be affected, once *E*[*Ẑ_R_*] begins to diminish (see *Eq. 17*). This performance loss begins for *R̂* > 0.5, in contrast to the performance of the continuous time model (see Fig. 7*B*).

When the robustness limit *R̂* > 0, we can again compute the two real roots of the associated MGF. Here, we use the increment distribution *f*_*Z*_*R*__(*Z*) given by *Eq. 7*, for which all probability mass within *R* of 0 is reassigned to 0. Surprisingly, upon plugging this distribution into the expression *M*_*Z*_(ω) = 1, we find that ω = 0, *h*_0_ continue to provide the two real solutions to this equation regardless of *R*, as depicted in Fig. 11*A*.

This observation implies that *1*) accuracies (*Eq. 16*) are unchanged as *R* is increased, and *2*) reaction times (*Eq. 17*) only change when *E*[*Z*_*R*_] changes. In other words, the integrator can ignore inputs below an arbitrary robustness limit at no cost to accuracy; the penalty will be incurred in terms of reaction time, and this will only be significant when *E*[*Z*_*R*_] changes appreciably. This result holds for any distribution for which:

(19) $$f_{Z}\left(z\right)=f_{Z}\left(-z\right)e^{-h_{0}z}.$$

it is straightforward to verify that the Gaussian satisfies this property (see *Derivation of Eq. 19* for more details).

How much of an increase in R is necessary to decrease *E*[*Z*_*R*_], the key quantity that alone controls performance loss? After substituting *R̂* = *R*/σ, we again find only one term up to fifth order in *R̂*,

(20) $$E\left[Z_{\hat{R}}\right]=E\left[Z\right]-\mu \sqrt{\frac{2}{9\pi }}e^{-\frac{1}{2}{\left(\frac{\mu }{\sigma }\right)}^{2}}{\hat{R}}^{3}+O\left({\hat{R}}^{5}\right)+\;\ldots \;,$$

indicating that modest amounts of robustness lead to only small changes in *E*[*Z*_*R̂*_]. [This is similar to the controlled duration case, where small values of *R̂* will have little effect on Accuracy*_R̂_*(*N*), cf. *Eq. 8*; see *Derivation of Eqs. 8 and 20* for more details.] This property, in combination with the constancy of *h*_0_, allows us to reason about the tradeoff of speed vs. accuracy under robustness. Under symmetrically bounded drift-diffusion, accuracy is determined by θ and *h*_0_, whereas the mean decision time is determined by θ, *h*_0_, and *E*[*Ẑ*]. Since *h*_0_ is fixed for all values of *R̂*, the predicted effect of robustness is a slowing of the decision time owing to the small change in *E*[*Z*_*R̂*_] (*Eq. 20*).

This effect is depicted in Fig. 10*B*. The solid curve is a locus of speed-accuracy combinations achieved by varying θ under no robustness. As in the previous section, the SNR of the independent sampling distribution is identical to the continuous time model (and performance at *R̂* = 0 is matched to Fig. 7*B* by varying the time intersampling time, here 37 ms). Performance begins to decrease at *R̂* = 0.5, and is much lower at *R̂* = 1 than the continuous time model with correlated evidence streams. As in the preceding section, robustness serves to decorrelate this input stream, effectively giving more independent samples and preserving performance beyond that predicted by the independent sampling theory.

#### Summary of analysis.

In the preceding three sections we have analyzed the impact of the robustness limit on decision performance. For both the controlled duration and reaction time tasks, we first studied the effect of this limit on the evidence carried by momentary values of sensory inputs. In each task, this effect was more favorable than might have been expected. In the controlled duration case, the SNR of momentary inputs was preserved for a fairly broad range of *R*, while in the reaction time task, *R* affected speed but not accuracy at fixed decision threshold. These results provided a partial explanation for the impact of robustness on decisions. The rest of the effect was attributed to the fact that the robustness mechanism serves to decorrelate input signals in time, further preserving decision performance by providing the equivalent of more independent evidence samples in a given time window.

### Reward Rate and the Robustness-Sensitivity Tradeoff

Up to now, we have examined performance in the reaction time task by plotting the full range of attainable speed and accuracy values. The advantage of this approach is that it demonstrates decision performance in a general way. An alternative, more compact approach, is to assume a specific method of combining speed and accuracy into a single performance metric. This approach is useful in quantifying decision performance and rapidly comparing a wide range of models.

Specifically, we use the reward rate (RR) (Gold and Shadlen 2002; Bogacz et al. 2006):

(21) $$\text{RR}=\frac{\text{Accuracy}}{\langle \text{RT}\rangle +T_{\text{del}}},$$

the number of correct responses made per unit time, where a delay *T*_del_ imposed between responses to penalizes rapid guessing. Implicitly, this assumes a motivation on the part of the subject that may not be true; in general, human subjects seldom achieve optimality under this definition as they tend to favor accuracy over speed in two-alternative forced choice trials (Zacksenhouse et al. 2010). Here, we simply use this quantity to formulate a scalar performance metric that provides a clear, compact interpretation of reaction time data.

**Fig. 12.**
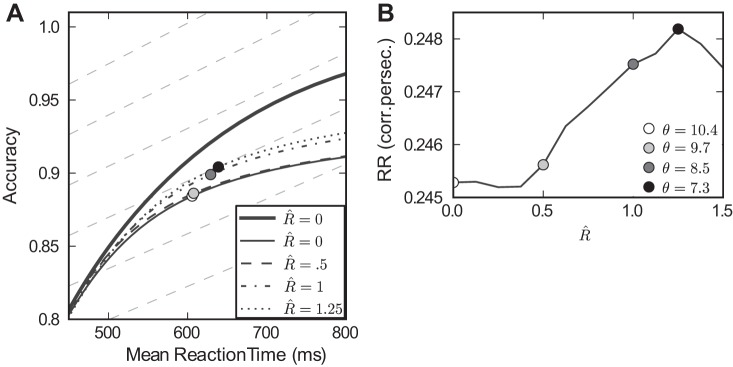
Robustness improves reward rate (RR) under mistuning. *A*: speed accuracy curves plotted for multiple values of *R̂*; as in previous figures, the greater accuracies found at fixed mean reaction times indicate that performance improves as *R̂* increases. The heavy line indicates the baseline case of a perfectly tuned, nonrobust integrator (repeated from Fig. 5*B*). RR level curves are plotted in background (dotted lines; see text), and points along speed accuracy curves that maximize RR are shown as circles. These maximal values of RR are plotted in *B*, demonstrating the nonmonotonic relationship between *R̂* and the best achievable RR.

Figure 12*A* shows accuracy vs. speed curves at four levels of *R̂*. The heavy solid line corresponds to the baseline model with robustness and mistuning set to zero (see Fig. 4). The lighter solid line corresponds to the mistuned model with σ_β_ = 0.1. The remaining broken lines correspond to the recovery model for three increasing levels of the robustness limit *R̂*. Also plotted in the background as dashed lines are reward rate level curves, that is, lines along which reward rate takes a constant value, with *T*_del_ = 3 s. On each accuracy vs. speed curve, there exists a reward rate-maximizing (reaction time, accuracy) pair. This corresponds to a tangency with one reward rate level curve and is plotted as a filled circle. In general, each model achieves maximal reward rate via a different threshold θ; values are specified in the legend of Fig. 12*B*. [A general treatment of reward rate-maximizing thresholds for drift-diffusion models is given in Bogacz et al. (2006).]

In sum, we see that mistuned integrators with a range of increasing robustness limits *R̂* achieve greater reward rate, as long as their thresholds are adjusted in concert. The optimal values of reward rate for a range of robustness limits *R̂* are plotted in Fig. 12*B*. Figure 12 illustrates the fundamental tradeoff between robustness and sensitivity. If there is variability in feedback mistuning (σ_β_ > 0), increasing *R̂* can help recover performance but only to a point. Beyond a certain level, increasing *R̂* further starts to diminish performance, as too much of the input signal is ignored. Overall, the impact on levels of reward rate is small: often ∼1%.

### Biased Mistuning Towards Leak or Excitation

**Fig. 13.**
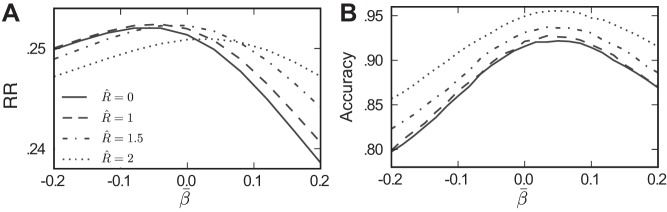
Robustness improves performance across a range of mistuning biases β̄. In both the reaction time (*A*) and controlled duration (*B*) tasks, robustness helps improve performance when β ∼ *N* (β̄, 0.1^2^), for all values of β̄ shown. As in previous figures, the coherence of the sensory input is *C* = 12.8. In the reaction time task (*A*), θ is varied for each value of β̄ to find the maximal possible RR, and performance gains are largest for β̄ > 0. In the controlled duration task, substantial gains are possible across the range of β̄ values.

We next consider the possibility that variation in mistuning from trial to trial could occur with a systematic bias in favor of either leak or excitation and ask whether the robustness limit has qualitatively similar effects on decision performance as for the unbiased case studied above. Specifically, we draw the mistuning parameter β from a Gaussian distribution with standard deviation σ_β_ = 0.1 as above but with various mean values β̄ (see materials and methods). In Fig. 13*A* we show reward rates as a function of the bias β̄, for several different levels of the robustness limit *R̂*. At each value of β̄, the highest reward rate is achieved for a value of *R̂* > 0; that is, regardless of the mistuning bias, there exists an *R̂* > 0 that will improve performance vs. the nonrobust case (R̂ = 0). We note that this improvement appears minimal for substantially negative mistuning biases (i.e., severe leaky integration) but is significant for the values of β̄ that yield the highest reward rate. Finally, the ordering of the curves in Fig. 13*A* shows that, for many values of β̄, this optimal robustness limit is an intermediate value less than *R̂* = 2.

While Fig. 13 only assesses performance via a particular performance rule (reward rate, *T*_del_ = 3 s), the analysis in *Reward Rate and the Robustness-Sensitivity Tradeoff* suggests that the result will hold for other performance metrics as well. Moreover, Fig. 13*B* demonstrates the analogous effect for the controlled duration task: for each mistuning bias β̄, decision accuracy increases over the range of robustness limits shown.

### Bounded Integration as a Model of the Fixed Duration Task

We have demonstrated that increasing the robustness limit *R̂* can improve performance for mistuned integrators, in both the reaction time and controlled duration tasks. In the latter, a decision was made by examining which integrator had accumulated more evidence at the end of the time interval.

In contrast, Kiani et al. (2008) argue that decisions in the controlled duration task may actually be made with a decision threshold, much like the reaction time task. That is, evidence accumulates until an absorbing bound is reached, causing the subject to ignore any further evidence and simply wait until the end of the trial to report the decision.

**Fig. 14.**
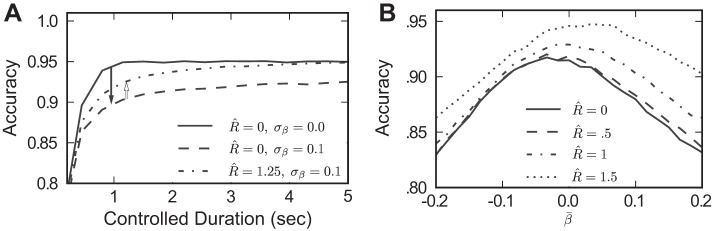
Effect of the robustness limit *R̂* on decision performance in a controlled duration task, under the bounded integration model of Kiani et al. (2008). Dot coherence *C* = 12.8. *A*: increasing the robustness limit *R̂* helps recover performance lost to mistuning at multiple reaction times in the controlled duration task. Specifically, moving from the baseline model to the mistuned model decreases decision accuracy, but this lost accuracy can be partially or fully recovered for *R̂* > 0. *B*: when allowing for biased mistuning (β̄ ≠ 0, σ = 0.1), *R̂* still allows for recovery of performance; effects are most pronounced when β̄ > 0.

Figure 14 demonstrates that our observations about how the robustness limit can recover performance lost to mistuned feedback carry over to this model of decision making as well. Specifically, Fig. 14*A* shows how setting *R̂* > 0 improves performance in a mistuned integrator. In fact, more of the lost performance is recovered than in the previous model of the controlled duration task (cf. Fig. 6*A*). Figure 14*B* extends this result to show that some value of *R̂* > 0 will recover lost performance over a wide range of mistuning biases β̄ (cf. Fig. 13*B*).

### Parameterizing and Comparing the Robust Integrator Model with Behavioral Data

We have demonstrated that robustness serves to protect an integrator against the hazards of runaway excitation and leak and that the cost of doing so is surprisingly small. Yet, it is hard to know whether the range of effects is compatible with known physiology and behavior. Without better knowledge of the actual neural mechanisms that support integration and decision making, it is not possible to directly reconcile the parameters of our analysis with physiology. However, in this study we required that the free parameter values in the models that we analyzed were compatible with measured psychophysics.

**Fig. 15.**
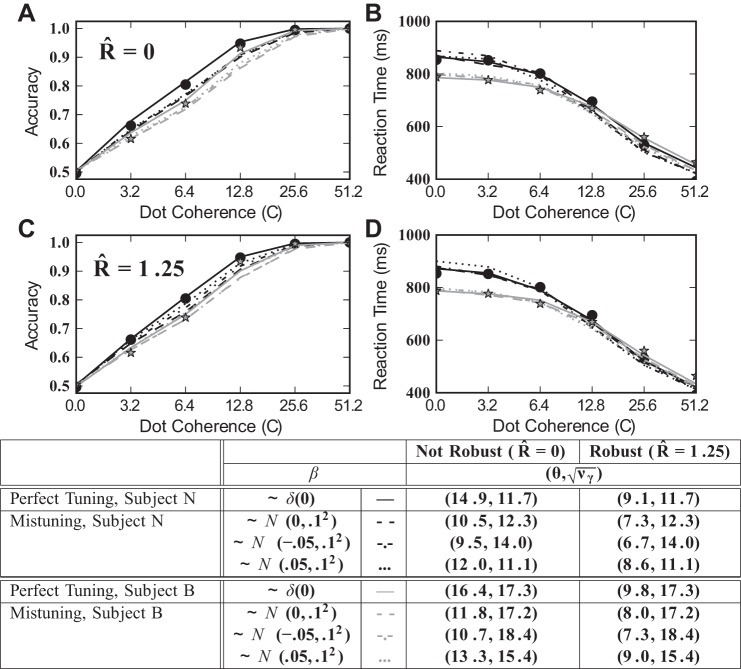
Accuracy (*A* and *C*) and chronometric (*B* and *D*) functions: data and model predictions. Solid dots and stars are behavioral data for a rhesus monkey [*subject* “*N*” and “*B*,” respectively (Roitman and Shadlen 2002)]. In *A–D*, the accuracy and chronometric functions are fit to behavioral data via least squares, over the free parameters θ and ν_γ_. In *A* and *B*, the robustness threshold *R̂* = 0, and results are shown for baseline and exemplar mistuned models (see legend in table). In *C* and *D*, results are shown for the robust and recovery models (*R* is fixed across the range of coherence values so that *R̂* = 1.25 at *C* = 0). The close matches to data points indicate that these models can be reconciled with the psychophysical performance of individual subjects by varying few parameters. Parameter values for each curve are summarized in the table.

To accomplish this, we fit accuracy and chronometric functions from robust integrator models to reaction time psychophysics data reported in Roitman and Shadlen (2002). This fit is via least squares across the range of coherence values and requires two free parameters for each integrator model: additive noise variance ν_γ_ and the decision bound θ (see materials and methods). Such noise and bound parameters are standard in fitting accumulator-type models to behavioral data. Figure 15 shows the results. Figure 15, *A* and *B*, displays accuracy and chronometric data together with fits for various integrator models, with *R̂* = 0. The solid line shows a close fit for the baseline model (i.e., with no feedback mistuning or robustness, see Fig. 4) to the behavioral data, in agreement with prior studies (Mazurek et al. 2003). The broken lines give analogous fits for mistuned models (σ_β_ = 0.1), with three values of bias in feedback mistuning (β̄).

Figure 15, *C* and *D*, shows the corresponding results for robust integrators. For all cases in these panels, we take the robustness limit *R̂* = 1.25. We fix levels of additive noise to values found for the nonrobust case above to demonstrate that by adjusting the decision threshold, one can obtain approximate fits to the same data. This is expected from our results above: Fig. 6 shows that, while accuracies at given reaction times are higher for mistuned robust vs. nonrobust models, the effect is modest on the scale of the full range of values traced over an accuracy curve. Moreover, for the perfectly tuned case, accuracies at given reaction times are very similar for robust and nonrobust integrators (Fig. 7, with a slightly lower value of *R̂*). Thus comparable pairs of accuracy and reaction time values are achieved for robust and nonrobust models, leading to similar matches with data. In sum, the accuracy and chronometric functions in Fig. 15 show that all of the models schematized in Fig. 4, baseline, mistuned, robust, and recovery, are generally compatible with the chronometric and accuracy functions reported in Roitman and Shadlen (2002).

A limitation of our analysis concerns the distribution of reaction time. Above we considered the effects of robustness on mean decision time but not on the shape of the distributions. The standard DDM predicts a longer tail to the reaction time distribution than is seen in data, thereby necessitating modifications to the simple model (Ditterich 2006; Ratcliff and Rounder 1998; Churchland et al. 2008). The most compelling modification in our view is a time-dependent reduction in the decision threshold θ. Our experience suggests that such modifications can also be implemented under robustness with and without mistuning. However, we did not attempt to fit the reaction time distributions from the experiments and leave the matter for future investigation.

## DISCUSSION

A wide range of cognitive functions require the brain to process information over time scales that are at least an order of magnitude greater than values supported by membrane time constants, synaptic integration, and the like. Integration of evidence in time, as occurs in simple perceptual decisions, is one such well-studied example, whereby evidence bearing on one or another alternative is gradually accumulated over time. This is formally modeled as a bounded random walk or drift diffusion process in which the state (or decision) variable is the accumulated evidence for one choice and against the alternative(s). Such formal models explain both the speed and accuracy of a variety of decision-making tasks studied in both humans and nonhuman primates (Ratcliff 1978; Luce 1986; Gold and Shadlen 2007; Palmer et al. 2005), and neural correlates have been identified in the firing rates of neurons in the parietal and prefrontal association cortex (Mazurek et al. 2003; Gold and Shadlen 2007; Churchland et al. 2008; Shadlen and Newsome 1996, 2001; Schall 2001; Kim et al. 2008). The obvious implication is that neurons must somehow integrate evidence supplied by the visual cortex, but there is mystery as to how.

This is a challenging problem because the biological building blocks operate on relatively short time scales. From a broad perspective, the challenge is to assemble neural circuits that that can sustain a stable level of activity (i.e., firing rate) and yet retain the capability to increase or decrease firing rate when perturbed with new input (e.g., momentary evidence). A well-known solution is to suppose that recurrent excitation might balance perfectly the decay modes of membranes and synapses (Cannon and Robinson 1983; Usher and McClelland 2001). However, this balance must be fine tuned (Seung 1996; Seung et al. 2000), or else the signal will either dissipate or grow exponentially (Fig. 1*A*, *top*). Several investigators have proposed biologically plausible mechanisms that mitigate somewhat the need for such fine tuning (Lisman et al. 1998; Goldman et al. 2003; Goldman 2009; Romo et al. 2003; Miller and Wang 2005; Koulakov et al. 2002). These are important theoretical advances because they link basic neural mechanism to an important element of cognition and thus provide grist for experiment.

Although they differ in important details, many of the proposed mechanisms can be depicted as if operating on a scalloped energy landscape with relatively stable (low energy) values, which are robust to noise and mistuning in that they require some activation energy to move the system to a larger or smaller value [Fig. 1*A*, *bottom*; cf. Pouget and Latham (2002); Goldman et al. (2009)]. The energy landscape is a convenient way to view such mechanisms, which we refer to as robust integrators, because it also draws attention to a potential cost. The very same effect that renders a location on the landscape stable also implies that the mechanism must ignore information in the incoming signal (i.e., evidence). Here, we have attempted to quantify the costs inherent in this loss. How much loss is tolerable before the circuit misses substantial information in the input? How much loss is consistent with known behavior and physiology?

We focused our analyses on a particular well-studied task because it offers critical benchmarks to assess both the potential costs of robustness to behavior and a gauge of the degree of robustness that might be required to mimic neurophysiological recordings with neural network models. Moreover, the key statistical properties of the signal and noise (to be accumulated) can be estimated from neural recordings.

We first imposed a modest amount of mistuning in recurrent excitation and asked whether robustness could protect against loss of decision-making performance. We found that it could. Although in general this protection is only partial (Figs. 6, 19), for the controlled duration task it can be nearly complete depending on the presence of a decision bound (Fig. 14*A*, controlled duration >3 s). In absolute terms, effect sizes are small for the task and mistuning levels that we modeled. A few percentage points of accuracy are lost due to mistuning and recovered due to robustness; this leads to only a small benefit in performance measures such as reward rate.

However, our most surprising result is not the subtle improvement that robustness can confer in our simulated task but the fact that levels of robustness can be quite high before they begin to degrade decision-making performance. Both in the presence and in the absence of mistuning, ignoring a large part of the motion evidence either slightly improved performance or produced an almost negligible decrement. This was the case even when more than a full standard deviation of the input distribution is ignored; in fact, this is the level of robustness that produced the best performance in the presence of mild mistuning.

We can appreciate the impact of robust integration by considering the distribution of random values that would increment the stochastic process of integrated evidence. Instead of imagining a scalloped energy surface, we simply replace all the small perturbations in integrated evidence with zeros. Put simply, if a standard integrator would undergo a small step in the positive or negative direction, a robust integrator instead stays exactly where it was. In the setting of drift diffusion, this is like removing a portion of the distribution of momentary evidence (the part that lies symmetrically about zero) and replacing the mass with a delta function at 0. At first glance this appears to be a dramatic effect, see the illustration of the distributions in Fig. 8, and it is surprising that it would not result in strong changes in accuracy or reaction time or both.

Three factors appear to mitigate this loss of momentary evidence. First, setting weak values of the input signal to zero can reduce both its mean and standard deviation by a similar amount, resulting in a small net change to the input SNR. Second, surprisingly, the small loss of signal-to-noise that does occur would not result in any loss of accuracy if the accumulation were to the same bound as for a standard integrator. The cost would be to decision time alone. Third, even this slowing is mitigated by the temporal dynamics of the input. Unlike for idealized drift diffusion processes, real input streams possess definite temporal correlation. Interestingly, removing the weakest momentary inputs reduces the temporal correlation of the noise component of the input stream. This can be thought of as allowing more independent samples in a given time period, thereby improving accuracy at a given response time.

While we used a simplified characterization of the robust integration operation in our study, we noted that there are many different ways in which this could be realized biologically (Koulakov et al. 2002; Nikitchenko and Koulakov 2008; Goldman et al. 2003, 2009; Fransén et al. 2006; Loewenstein and Sompolinsky 2003; Egorov et al. 2002). In appendix a, we make this connection concrete, for one such mechanism based on bistable neural pools. An intriguing finding presented there (see Fig. ) is that the robustness mechanism provided by the circuit-based bistable model produces an even more favorable effect of robustness for decision making than the simplified model in the main text. This demonstrates the generality of our results and points to an intriguing area of future study, focusing on the impact of more detailed circuit- and cell-level dynamics.

Our robust integrator framework shares features with existing models in sensory discrimination. The interval of uncertainty model of Smith and Vickers (1989) and the gating model of Purcell et al. (2010) ignore part of the incoming evidence stream, yet they can explain both behavioral and neural data. We suspect that the analyses developed here might also reveal favorable properties of these models. Notably, some early theories of signal detection also featured a threshold, below which weaker inputs fail to be registered, the so-called high threshold theory (reviewed in Swets 1961). The primary difference in the current work is to consider single decisions made based on an accumulation of many such thresholded samples (or a continuous stream of them).

Although they are presented at a general level, our analyses make testable predictions. For example, they predict that pulses of motion evidence added to random dot stimulus would affect decisions in a nonlinear fashion consistent with a soft threshold. Such pulses are known to affect decisions in a manner consistent with bounded drift diffusion (Huk and Shadlen 2005) and its implementation in a recurrent network (Wong et al. 2008). A robust integration mechanism further predicts that brief, stronger pulses will have greater impact on decision accuracy than longer, weaker pulses containing the same total evidence. Beyond pulses with different characteristics, it is possible that an analogous thresholding effect could be seen for periods of strong and weak motion evidence in the random dots stimuli themselves, although this would require further study to assess.

However, we believe that the most exciting application of our findings will be to cases in which the strength of evidence changes over time, as expected in almost any natural setting. One simple example is for task stimuli that have an unpredictable onset time and whose onset is not immediately obvious. For example, in the moving dots task, this would correspond to subtle increases in coherence from a baseline of zero coherence. Our preliminary calculations agree with intuition that robust integrator mechanism will improve performance: in the period before the onset of coherence, less baseline noise would be accumulated; after the onset of coherence, the present results suggest that inputs will be processed with minimal loss to decision performance. This intuition can be generalized to apply to a variety of settings with nonstationary sensory streams.

Many cognitive functions evolve over time scales that are much longer than the perceptual decisions we consider in this study. Although we have focused on neural integration, it seems likely that many other neural mechanisms are also prone to drift and instability. Hence, the need for robustness may be more general. Yet, it is difficult to see how any mechanism can achieve robustness without ignoring information. If so, our finding may provide some optimism. Although we would not propose that ignorance is bliss, it may be less costly than one would expect.

## GRANTS

This research was supported by a Career Award at the Scientific Interface from the Burroughs-Wellcome Fund (to E. Shea-Brown), Howard Hughes Medical Institute, the National Eye Institute Grant EY-11378, and National Center for Research Resources Grant RR-00166 (to M. Shadlen), a seed grant from the Northwest Center for Neural Engineering (to E. Shea-Brown and M. Shadlen), NSF Teragrid Allocation TG-IBN090004, and in part by the University of Washington eScience Institute.

## DISCLOSURES

No conflicts of interest, financial or otherwise, are declared by the author(s).

## AUTHOR CONTRIBUTIONS

Author contributions: E.S.-B., N.C., A.K.B., and M.S. conception and design of research; E.S.-B., N.C., A.K.B., and M.S. performed experiments; E.S.-B., N.C., A.K.B., and M.S. analyzed data; E.S.-B., N.C., A.K.B., and M.S. interpreted results of experiments; E.S.-B., N.C., A.K.B., and M.S. prepared figures; E.S.-B., N.C., A.K.B., and M.S. drafted manuscript; E.S.-B., N.C., A.K.B., and M.S. edited and revised manuscript; E.S.-B., A.K.B., and M.S. approved final version of manuscript.

## Figures and Tables

**Fig. A1.**
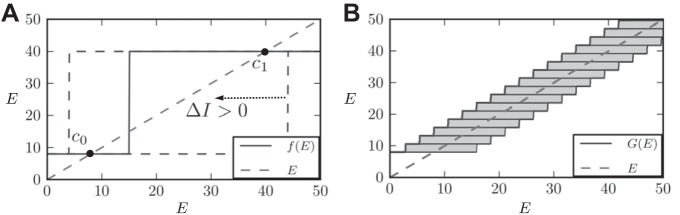
Simultaneous plots of the identity line and the feedback line, *G*(*Ê*), for 2 circuits with differing numbers of subunits. *A*: here *N* = 1, and so the feedback line *G*(*Ê*) = *f*(*Ê*) is exactly determined as a function of *Ê* = *r*_1_ (see *Eq. 25*). The 2 intersections *c*_0_ and *c*_1_ are stable fixed points. In this way, the subunit firing rate is bistable, and the value attained will depend on the history of the circuit activity. As Δ*I* is changed, this translates *f*(*Ê*); eventually eliminating either *c*_0_ or *c*_1_ and forcing the subunit to the remaining stable fixed point (here, *c*_0_ corresponds to *Ê* = 8 Hz. and *c*_1_ to 40 Hz). In dashed curves, the feedback line is plotted for 2 such values of Δ*I. B*: now *N* > 1 and so the feedback line *G*(*Ê*) is no longer unambiguously specified as a function of its argument. The function is instead the sum of *N* potentially bivalued functions, whose actual values will depend on the stimulus history. We represent this fact by plotting the feedback line as a set of stacked boxes, representing the potential contribution of the *i*th subunit to the total integrator dynamics (Goldman et al. 2003).

**Fig. A2.**
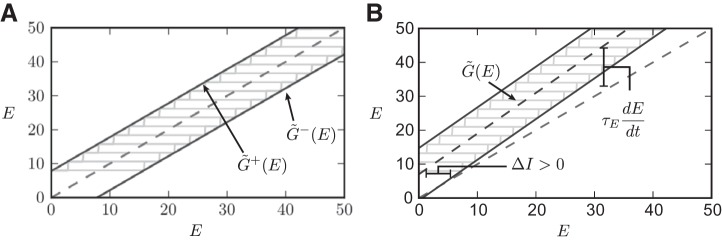
Plot of possible equilibria for *Eq. 24. A*: extent of each multivalued feedback function defines the minimum input necessary to perturb the system away from equilibrium, defining the fixation lines. As *N* → ∞, the stable fixed points become more tightly packed on the interval (*r*^−^; *r*^+^). *B*: when the integrator is mistuned, the fixation lines and the feedback line are no longer parallel. The rate that the integrator accumulates input is approximated by the distance between the center line of the feedback subunits [*G̃*(*Ê*)], and the feedback line. However, integration only occurs when the “fixation condition” is no longer satisfied, i.e., when the feedback line is no longer bounded by the fixation lines at the current value of *Ê*.

**Fig. A3.**
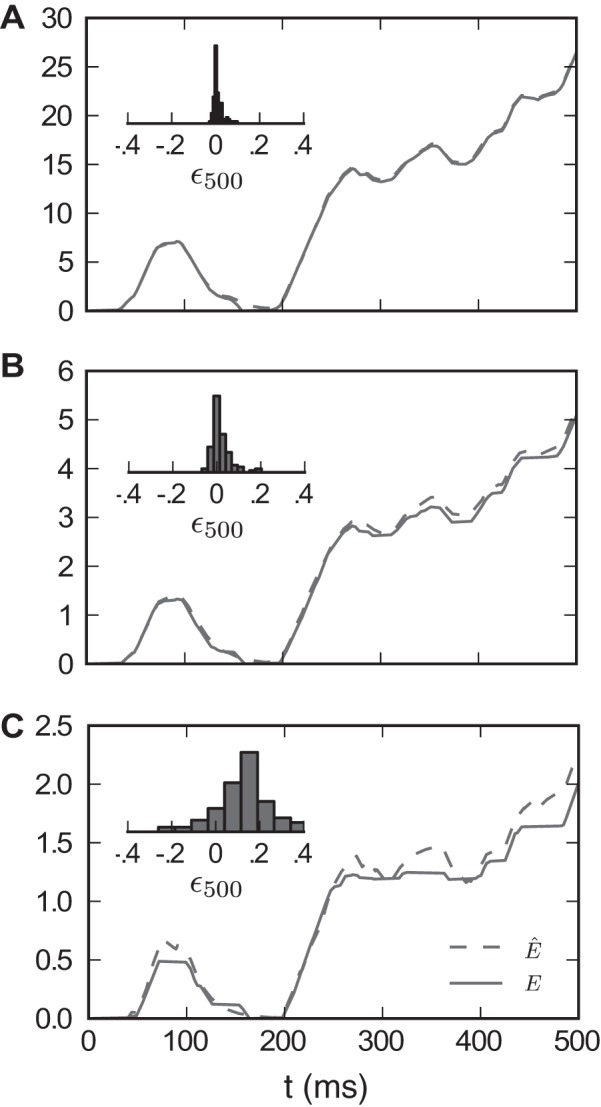
Comparison of integration by *Eqs. 22* and *5*, *R̂* = 0.1 (*A*), *R̂* = 0.5 (*B*), and *R̂* = 11 (*C*). Histograms of the relative error between the values of *E*(*t*) and *Ê*(*t*) at *t* = 500 ms are plotted as *insets* (see *Eq. 36*). Means and SD (μ, σ) for each distribution are, respectively, (0.0097, 0.022), (0.0183, 0.0453), and (0.1342, 0.1358). At these levels of *R̂*, *E* approximates *Ê* low to moderate error.

## APPENDIX A: A DERIVATION OF THE ABSTRACTED ROBUST INTEGRATOR MODEL FROM THE DYNAMICS OF NEURAL SUBUINITS

In this section, we demonstrate one method to construct a circuit-based robust integrator with the properties described in Fig. 1 and described by the abstracted integrator model of the main text (*Eq. 5*). We emphasize that this is not the only possible way that these integrator dynamics could be implemented and that the aim of the below is to provide a single, concrete example. For related approaches see Koulakov et al. (2002) and Nikitchenko and Koulakov (2008) and also the behavior of single-cell integrators that are insensitive to weak fluctuations [for example, see Egorov et al. (2002) and Fransén et al. (2006)].

Our construction follows closely that of Koulakov et al. (2002) and Goldman et al. (2003). Accordingly, robustness arises from the bistability of multiple self-excitatory subpopulations (or subunits). The dynamics of each subunit are governed by one of *N* differential equations. Depending on the activity in the rest of the network, each individual subunit can be become bistable, so that its eventual steady-state value (i.e., “On” or “Off”) depends on its past. This effect, known as hysteresis, underlies several robust integrator models (Koulakov et al. 2002; Goldman et al. 2003).

The circuit integrates inputs via sequential activation of subunits, in an order determined by graded levels of “background” inputs (or biases) to each subunit. Following Goldman et al. (2003), we collapse the *N* differential equations that describe individual subunits into an equation that approximates the dynamics of the entire integrator. This expression for the total firing rate *Ê*(*t*) averaged over all subpopulations reduces to the robust integrator analyzed in the main text (*Eq. 7*).

### Firing Rate Model

The firing rate *r*_*i*_(*t*) of the *i*th bistable subunit (*i* ∈ {1, 2,…, *N*}) is modeled by a firing rate equation:

(22) $$\tau_{E}\frac{dr_{i}}{dt}=-r_{i}+r^{-}+\left(r^{+}-r^{-}\right)H\left[pr_{i}+q\left(1+\beta \right)\sum_{i\neq j}^{N}r_{j}+a\Delta I-b_{i}\right]$$

(23) $$\hat{E}=\frac{1}{N}\sum_{i=1}^{N}r_{i}$$

Figure A1 demonstrates the firing rate dynamics of a circuit composed of a single subunit (*N* = 1). We plot the identity line, corresponding to the first “decay” term in *Eq. 22*, and the “feedback” line, corresponding to the second. Since *N* = 1, this simplifies to *f*(*r*_1_). The two intersections marked *c*_0_ and *c*_1_ are stable fixed points (which we refer to as On and Off, respectively). Thus the subunit shown is bistable. Importantly, however, the location of the step in *f*(*Ê*) varies with changes in the input signal (as per *Eq. 22*). In particular, substantial values of Δ*I*(*t*) will (perhaps transiently) eliminate one of the fixed points, forcing the subunit into either the On or the Off state with *r*_*i*_ = *r*_+_ or *r*_*i*_ = *r*_−_, respectively. Moreover, the change is self-reinforcing via the recurrent excitation *pr*_*i*_. The range over which a given subunit displays bistability is affected by the mistuning parameter β, which scales the total recurrent excitation from the rest of the circuit.

The firing rate dynamics, *Ê*(*t*), are obtained by summing both sides of *Eq. 22* over

(24) $$\tau_{E}\frac{d\hat{E}}{dt}=-\hat{E}+G\left(\hat{E}\right)$$

where

(25) $$G\left(\hat{E}\right)=r^{-}+\frac{\left(r^{+}-r^{-}\right)}{N}\sum_{i=1}^{N}H\left[\left(p-q\left(1+\beta \right)\right)r_{i}+N_{q}\left(1+\beta \right)\hat{E}+a\Delta \text{I}-b_{i}\right].$$

At this point, we almost have a differential equation for a single variable, *Ê*(*t*). However, *Eq. 25* still depends on the *N* activities *r_i_* of the individual subunits, and at any particular time their values are not uniquely determined by the value of *Ê*; we can only bound their values as *r*^−^ ≤ *r*_*i*_ ≤ *r*^+^.

### Bias Term

The bias term for the *i*th subunit, *b*_*i*_, is set by determining the range of values of *Ê* for which the exact value of the feedback function *f*(*r*_*i*_) is unknown. In the case of an integrator composed of only a single subunit, the bias term causes the positive input needed to force the unit to be on, and the negative input needed to force the unit to be off, to take the same values. This yields *b*_1_ = *p*(*r*^+^ + *r*^−^)/2.

The general case of *N* subunits is more complicated. Now the feedback contribution of the *i*th unit, *f*(*r*_*i*_), is no longer a simple function of the population activity *Ê*. Instead, it has additional dependence on its own activity *r*_*i*_. We see this clearly in *Eq. 25*, where the values of *r*_*i*_ that contribute to the definition of *G*(*Ê*) are unspecified. However, we do know that each *r_i_* is trapped between *r*^+^ and *r*^−^. Therefore, we can plot *G*(*Ê*) as the sum of a sequence of bivalued functions of *Ê*; see Fig. 16*B* and Goldman et al. (2003). The contribution from each pool is then represented by the shaded region. Finally, the bias terms are chosen to center these shaded boxes over the identity line:

(26) $$b_{i}=p\left(\frac{r^{-}+r^{+}}{2}\right)+q\left(\left(i-1\right)r^{+}+\left(N-i\right)r^{-}\right).$$

### Fixation Lines

We next define the “fixation” lines [*G̃*^+^(*Ê*) and *G̃*^−^(*Ê*)], which are the consequence of the multivalued property of the integrator. These lines define a region (the fixation region) that runs across the outermost corners of the “stacked boxes” in Fig. A2.

(27) $${\tilde{G}}^{+}\left(\hat{E}\right)=\hat{E}\left(1+\beta \right)+\frac{a\Delta I}{Nq}-\frac{\beta r^{+}}{N}+\frac{p\left(r^{+}-r^{-}\right)}{2Nq}$$

(28) $${\tilde{G}}^{-}\left(\hat{E}\right)=\hat{E}\left(1+\beta \right)+\frac{a\Delta I}{Nq}-\frac{\beta r^{-}}{N}+\frac{p\left(r^{+}-r^{-}\right)}{2Nq}$$

The term fixation region refers to the following property: if the input Δ*I* is such that the identity line lies within the fixation region, then the integrator will possess a range of closely spaced fixed points [where *Ê* = G(*Ê*)]. Thus *E* is not expected to change from its current value and integration of Δ*I* will not occur. Recall that Δ*I* acts to shift these boxes leftward or rightward relative to the identity line, just as in the analysis of Fig. A1. As a consequence, it is weak inputs that fail to be integrated.

From this analysis, we can see that integration by the system as a whole relies on two concepts. The first is a condition on Δ*I* necessary to eliminate fixed points; we call this the “fixation condition.” The second is the question of how quickly to integrate once this condition is no longer satisfied.

### Integration

Based on the analysis above, we derive a reduced model that approximately captures the dynamics of the “full” model indicated by *Eq. 24*. We call this the “effective” model. The rate of change of *Ê*, i.e., the rate of integration, is given by the distance between the current value of *G*(*Ê*) and the identity line. We approximate this by the distance between the middle of the fixation lines, which we define as *G̃*(*Ê*), and the identity line. This is pictured in Fig. A2 and yields:

(29) $$\tau_{E}\frac{d\hat{E}}{dt}=-\hat{E}+G\left(\hat{E}\right)\approx -\hat{E}+\tilde{G}\left(\hat{E}\right)$$

(30) $$\tilde{G}\left(\hat{E}\right)=\left(1+\beta \right)\hat{E}+\frac{a\Delta I}{Nq}-\beta \frac{\left(r^{+}-r^{-}\right)}{2N}$$

We emphasize that integration by this equation only occurs when the fixation condition is no longer satisfied, i.e., when the fixation lines no longer bound the identity line.

### Fixation Condition

The last step in defining the one-dimensional effective model is determining the fixation condition. We must solve for the values of *Ê* that cause the feedback line to lie between the two fixation lines:

(31) $$\text{No}\;\text{change}\;\text{in}\;\hat{E}\Leftrightarrow {\tilde{G}}^{-}\left(\hat{E}\right)<\hat{E}<{\tilde{G}}^{+}\left(\hat{E}\right)$$

(32) $$\Leftrightarrow \tilde{G}\left(\hat{E}\right)-\frac{\left(r^{+}-r^{-}\right)\left(p-q\beta \right)}{2\text{N}q}<\hat{E}<\tilde{G}\left(\hat{E}\right)+\frac{\left(r^{+}-r^{-}\right)\left(p-q\beta \right)}{2Nq}$$

(33) $$\Leftrightarrow \left|\beta \hat{E}+\frac{a\Delta I}{Na}-\beta \frac{\left(r^{+}+r^{-}\right)}{2N}\right|<\frac{\left(r^{+}-r^{-}\right)\left(p-q\beta \right)}{2Nq}$$

If this condition is violated with Δ*I* = 0, the integrator displays runaway integration (β > 0) or leak (β < 0). If it is satisfied when Δ*I* = 0, we have a condition on the level of Δ*I* that must be present for integration to occur. This yields a piecewise-defined differential equation, corresponding to when integration can and cannot occur:

(34) $$\tau_{E}\frac{d\hat{E}}{dt}\approx \{\begin{array}{ll} 0 & \left|\beta \hat{E}+\frac{a\Delta I}{Nq}-\beta \frac{\left(r^{+}-r^{-}\right)}{\text{2}N}\right|<\frac{\left(r^{+}-r^{-}\right)\left(p-\beta q\right)}{\text{2}Nq}\\ \beta \hat{E}+\frac{a\Delta I}{Nq}-\beta \frac{\left(r^{+}-r^{-}\right)}{\text{2}N} & \text{otherwise} \end{array}$$

We now simplify this equation; we assume that *p* ∼ *O*(*N*) and *a* ∼ *O*(*N*), i.e., the local feedback term and input weight can be increased as *N* is increased. With all other terms held constant, we relabel κ = *a*/*Nq* and *R* = (*r*^+^ − *r*^−^)*p*/2*a*; here *R* is the robustness parameter in the main text, and can be decreased or increased by adjusting *p*. This yields the central relationship of the main text:

(35) $$\tau_{E}\frac{dE}{dt}=\{\begin{array}{ll} 0 & \left|\beta E+\kappa \Delta I\right|\leq \kappa R\\ \beta E+\kappa \Delta I & \text{otherwise} \end{array}$$

Figure A3 compares the integrators defined by *Eqs. 22* and *35* at three different values of the normalized robustness limit *R̂*, in response to a fixed realization of Δ*I*(*t*) for comparison. As *R̂* increases, the extent to which the effective model tracks the full model decreases. The quality of the reduction can be quantified by examining the relative error between the full and effective models:

(36) $$\varepsilon_{t}=\frac{\hat{E}\left(t\right)-E\left(t\right)}{\hat{E}\left(t\right)}$$

Histograms of ε_*t*_ evaluated at *t* = 500 ms are included in Fig. A3, *insets*. The average agreement of the two models is within roughly 20% across a range of robustness values *R̂*. This agreement on the basis of individual trials is sufficient for the purpose of demonstrating the connection between the simplified integrator model we analyze, and one of its many possible neural substrates. A performance comparison between these models is included in Fig. A4, *top*.

## APPENDIX B: ADDITIONAL MATHEMATICAL DETAILS

### Derivation of Eq. 19

In this section we derive a necessary and sufficient condition on the PDF of a random variable (RV) in order for a key property to hold. This is that the nontrivial real value where its MGF is equal to one does not change, when its PDF is modified so that samples with absolute value less than a given threshold are set to zero. More concretely, let *X* be a RV with PDF *f*(*x*), and let *h*_0_ be value in question for the MGF of *X*:

(37) $$\int_{-\infty }^{\infty }f\left(x\right)e^{h_{0}*x}dx=1.$$

We next define a new random variable *X̂* with a PDF *f̂*(*x*) constructed by reallocating probability mass below a threshold *R* to a weighted delta function at zero:

(38) $$\hat{f}\left(x\right)=\delta \left(x\right)\int_{-R}^{R}f\left(x\prime \right)dx\prime +\{\begin{array}{ll} 0 & \left|x\right|<R\\ f\left(x\right) & \text{otherwise} \end{array}.$$

Then we propose that the following condition:

(39) $$f\left(-x\right)=f\left(x\right)e^{h_{0}x}$$

is necessary and sufficient to conclude:

(40) $$\int_{-\infty }^{\infty }\hat{f}\left(x\right)e^{h_{0}*x}dx=1,\;\text{for}\;\text{any}\;R>0$$

i.e., that the value *h*_0_ where the MGF is equal to one remains unchanged for any positive value of the threshold *R*.

To begin, we rewrite the left-hand side of *Eq. 40* as:

(41) $$\int_{-\infty }^{\infty }\hat{f}\left(x\right)e^{h_{0}*x}dx=\int_{-\infty }^{-R}f\left(x\right)e^{h_{0}*x}dx+\int_{R}^{\infty }f\left(x\right)e^{h_{0}*x}dx+\int_{-R}^{R}f\left(x\right)dx.$$

Sufficiency then follows via direct substitution of *Eq. 39* into the last expression on right-hand side.

More interesting is demonstrating that *Eq. 39* is necessary to conclude the key relationship of *Eq. 40*. We accomplish this by adding in, and subtracting back out, the integral over the “central” part of the real number line:

(42) $$1=\int_{-\infty }^{\infty }\hat{f}\left(x\right)e^{h_{0}*x}dx$$

(43) $$=\int_{-\infty }^{-R}f\left(x\right)e^{h_{0}*x}dx+\int_{R}^{\infty }f\left(x\right)e^{h_{0}*x}dx+\int_{-R}^{R}f\left(x\right)dx$$

(44) $$+\left(\int_{-R}^{R}f\left(x\right)e^{h_{0}*x}dx-\int_{-R}^{R}f\left(x\right)e^{h_{0}*x}dx\right)$$

Subtracting 1 from each part of this equation, this implies:

(45) $$0=\int_{-R}^{R}f\left(x\right)-f\left(x\right)e^{h_{0}*x}dx$$

Let *g*(*x*) = *f*(*x*) − *f*(*x*)*e*^*h*_0_^*^*x*^; it is clear then that *Eq. 45* holding for all *R* is equivalent to *g*(*x*) being either identically zero, or an odd function (so long as the expression must hold for all *R*). Now *g*(*x*) = 0 could require that *f*(*x*) = 0, which is impossible given that *f*(*x*) is a PDF; therefore, *g*(*x*) must be an odd function:

(46) −g(x)=g(−x)

(47) $$\Leftrightarrow -\left(f\left(x\right)-f\left(x\right)e^{h_{0}*x}\right)=f\left(-x\right)-f\left(-x\right)e^{-h_{0}*x}$$

(48) $$\Leftrightarrow -f\left(x\right)\left(1-e^{h_{0}*x}\right)=f\left(-x\right)\left(1-e^{-h_{0}*x}\right)$$

(49) $$\Leftrightarrow -f\left(x\right)\frac{\left(1-e^{h_{0}*x}\right)}{\left(1-e^{-h_{0}*x}\right)}=f\left(-x\right)$$

(50) $$\Leftrightarrow f\left(x\right)e^{h_{0}*x}=f\left(-x\right)$$

Thus *Eq. 39* is also a necessary condition for *Eq. 40*.

### Derivation of Eqs. 8 and 20

*Equation 8* is a Taylor expansion in *R* of the accuracy formula for a sum of *N* IID random variables. Assuming that this sum, *S* = ∑_*i*=1_^*N*^*X*_*i*_, can be approximated with a Gaussian, this formula is:

(51) $$\text{Accuracy}\approx \frac{1}{\sqrt{2\pi \text{Var}\left[S\right]}}\int_{0}^{\infty }e^{-\frac{{\left(x-E\left[S\right]\right)}^{2}}{2\text{Var}\left[S\right]}}dx.$$

Assuming that *X*_*i*_ has a PDF given by a Gaussian with probability mass below a threshold *R* reallocated to 0 (see main text, and also *Derivation of Eq. 19*), the expectation and variance of *S* can be computed as:

(52) $$E\left[S\right]=N\left(s\frac{1}{2}\text{Erf}\left(\frac{s-R}{\sqrt{2}}\right)-s\frac{1}{2}\text{Erf}\left(\frac{R+s}{\sqrt{2}}\right)+\frac{e^{-\frac{1}{2}{\left(R-s\right)}^{2}}}{\sqrt{2\pi }}-\frac{e^{-\frac{1}{2}{\left(R+s\right)}^{2}}}{\sqrt{2\pi }}+s\right)$$

(53) Var[S]=N2πe−12(R+s)2(π(s2+1)e12(R+s)2Erf(s−R2)−π(s2+1)e12(R+s)2Erf(R+s2)+2πs2e12(R+s)2+2se2Rs+2Re2Rs+2πe12(R+s)2+2R−2s)−NE[S]2

Expanding *Eq. 52* in *R* about *R* = 0 yields *Eq. 20*, while combining these formulas into *Eq. 51* and expanding gives *Eq. 8*.
